# Gastric mixed neuroendocrine non-neuroendocrine neoplasms

**DOI:** 10.3389/fonc.2024.1335760

**Published:** 2024-04-08

**Authors:** Li Liu, Qian Li, Wenxuan Liu, Zhendong Qiu, Zhongkai Wu, Danli Yu, Wenhong Deng

**Affiliations:** ^1^ Department of General Surgery, Renmin Hospital of Wuhan University, Wuhan, Hubei, China; ^2^ Department of Ultrasound Imaging, Hubei Cancer Hospital, Tongji Medical College, Huazhong University of Science and Technology, Wuhan, Hubei, China

**Keywords:** MINENs, gastric cancer, neuroendocrine tumours, treatment, prognosis

## Abstract

The uncommon tumour known as gastric mixed neuroendocrine-non-neuroendocrine neoplasms (G-MiNENs) is made up of parts of neuroendocrine carcinoma and adenocarcinoma. The biological and clinical features are different from those of gastric adenocarcinoma. Their pathophysiology, diagnostic standards, and clinical behaviour have all been the subject of lengthy debates, and their nomenclature has undergone multiple changes. Its emergence has created new challenges in the classification and diagnosis of gastric tumours. This review will update information on the topic, covering molecular aspects, diagnostic criteria, treatment, and prognostic factor discovery. It will also provide a historical context that will aid in understanding the evolution of the idea and nomenclature of mixed gastric tumours. Additionally, it will provide the reader a thorough understanding of this difficult topic of cancer that is applicable to real-world situations.

## Introduction

Rare malignant tumours are known as mixed neuroendocrine-non-neuroendocrine neoplasms (MiNENs). A subgroup of gastric tumours known as g-MiNENs, or gastric mixed neuroendocrine-non-neuroendocrine neoplasms, is morphologically diverse and clinically aggressive. Most individuals with MiNENs are older than 50 years of age, and most MiNENs are discovered at an advanced stage of illness (1). As awareness of MiNEN increases and diagnostic techniques improve, the number of patients tends to increase (2).

The emergence of MiNEN has brought new challenges in the classification and diagnosis of gastric tumours. G-MiNENs are usually large, polypoid, ulcerated or stenotic lesions (3), with a male predominance in g-MINENs (4). MiNENs can occur throughout the body, but most are located in the gastrointestinal tract (5), especially the stomach (6–8). In a large cohort study of 1857 cases of gastroenteropancreatic neuroendocrine neoplasm (GEP-NEN), a total of 129 cases were diagnosed with MINENs, with the highest proportion of primary lesions being in the stomach (78 cases, 60.5%). The number and percentage of patients with other focal sites were: colorectal (19, 14.7%), oesophageal (13, 10.1%), duodenal (5, 3.9%), biliary (4, 3.1%), pancreatic (4, 3.1%) and other sites (6, 4.7%) (9). In addition, compared to other digestive tract organs, the stomach had a lower rate of MiNENs survival (10). G-MiNENs remain a rare tumour, accounting for less than 1% of all gastric tumours (4, 11), In a study that included 3961 patients after gastrectomy for gastric cancer, pathological findings showed that there were only 14 cases of g-MINENs, accounting for 0.35% of gastric cancer resections, in which the prevalence of MINENs was significantly higher in the gastro-oesophageal junction than in other regions of the stomach (12). Given that g-MiNEN is rare and heterogeneous, questions about their pathogenesis, diagnostic criteria, treatment and prognosis have been a matter of debate for many years.

This study aims to clarify what is meant by gastric mixed neuroendocrine-non-neuroendocrine neoplasms, summarize current methods for diagnosing them histopathologically and for determining the molecular evidence pertaining to their pathophysiology, and discuss current methods for treating them. This paper assesses the clinicopathological characteristics and therapeutic advancements in patients with g-MiNENs by reviewing recent studies and reports on the condition. The aim is to make continuous progress in the understanding and treatment of the disease, leading to better survival and prognosis for patients.

## Definition of MiNENs

Mixed neuroendocrine-non-neuroendocrine neoplasms are neuroendocrine neoplasms (NENs) that contain both neuroendocrine (NE) and non-neuroendocrine (NNE) components, with at least 30% of each component (13). In the stomach, the NNE component of more than 90% of cases consists of adenocarcinomas and rarely other epithelial tumours, including imprinted cell carcinoma and squamous cell carcinoma (14).

MiNENs are a specific type of NENs. NENs are a class of tumours originating from peptidergic neurons and neuroendocrine cells that have neuroendocrine differentiation and express neuroendocrine markers (15). The histological differentiation of NENs is categorized into two groups: poorly differentiated neuroendocrine carcinomas (NECs) and well-differentiated neuroendocrine tumours (NETs).The neuroendocrine component of the majority of g-MiNENs consists of NECs, and rarely NETs (16). Adenocarcinomas made up the majority of the g-MINEN exocrine components observed, while NECs made up the majority of the NE components (17). G-MiNENs, which consist of both neuroendocrine and non-neuroendocrine components, are uncommon, making up approximately 7% of all gastric neuroendocrine tumours and 25% of all gastric poorly differentiated neuroendocrine carcinomas (18).

**Figure 1:**
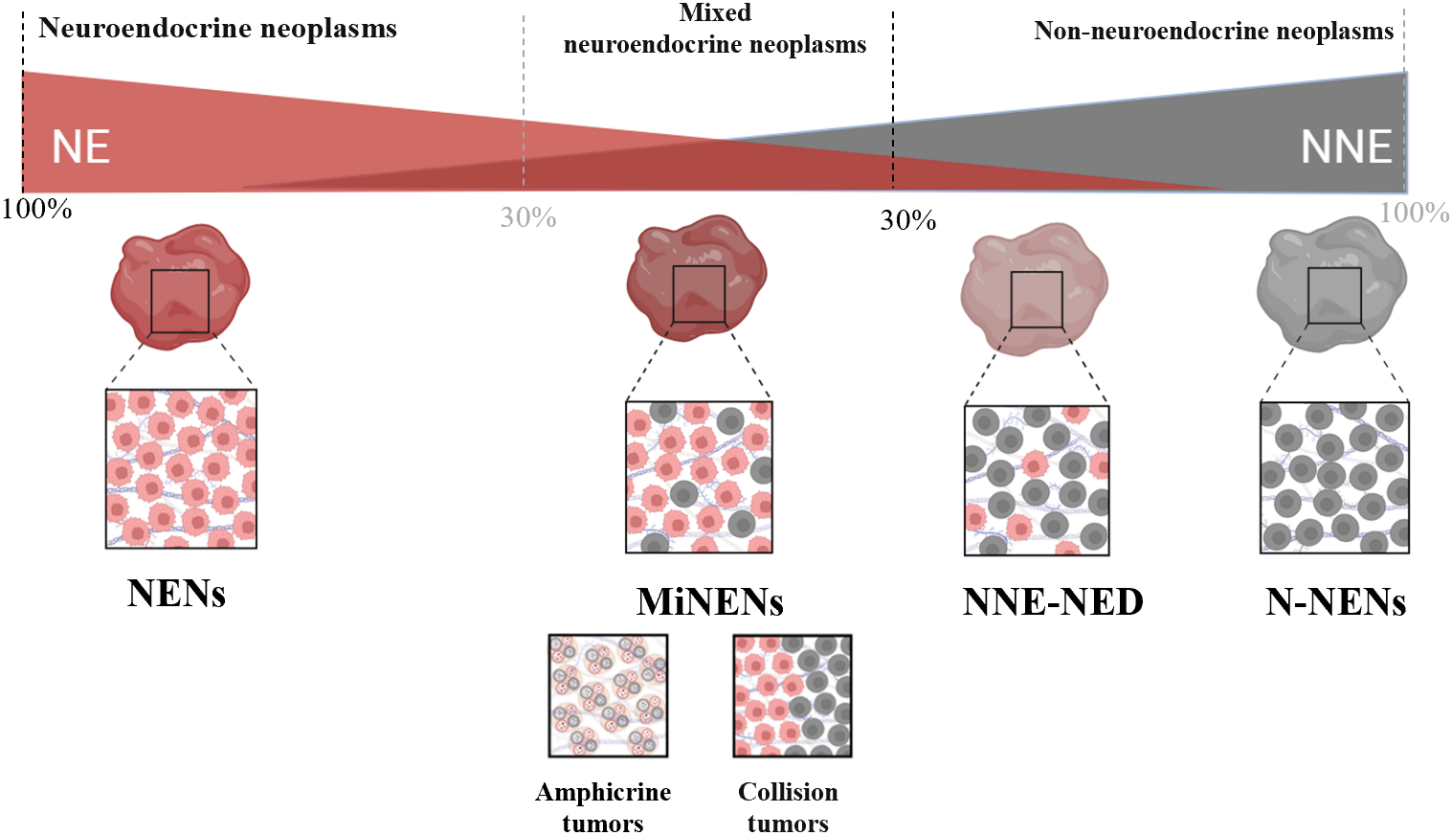
Classification system for mixed neuroendocrine tumours.

It is important to note that MiNENs have a certain scope of application. Lewin (19) first established a comprehensive classification scheme for mixed neuroendocrine tumours in 1987, referring to what we now refer to as MiNENs as “composite glandular-endocrine cell carcinomas”. His classification scheme separates these tumours from non-NE tumours with less than 30% NE differentiation, as well as from collision tumours and amphicrine tumours. A particular subtype of mixed NENs is called MiNENs (**Figure 1**). In general, the morphology of MiNENs is expressed as a composite tumour since both NE and NNE components are closely intermingled and originate from a single common precursor cell. Two distinct primary tumours that have unintentionally developed into the same area are what make up collision tumours. Every tumour begins as a distinct progenitor cell that has undergone autonomous molecular evolution (20). Each sort of single cell that makes up an amphicrine tumour has both NE and NNE characteristics. Mucus vesicles and neurosecretory granules, for instance, can be seen in the same cell using electron microscopy. While they are somewhat more common in the pancreas, these tumours are incredibly uncommon in the stomach (21). Non-neuroendocrine tumours with neuroendocrine differentiation (NNE-NED) are non-NE tumours that exhibit NE differentiation, but not enough of it to be classified as a MiNENs. It is important to acknowledge that the present threshold of 30% was initially established in 1987 in an arbitrary manner. Its purpose was to delineate and establish a distinct diagnostic category for tumours exhibiting a significant proportion of both non-neuroendocrine and NE components. Additionally, this threshold aimed to exclude tumours lacking NE characteristics but containing scattered NE cells whose clinical relevance remained uncertain (22). Despite its significant contribution to the recognition of MiNENs as separate diagnostic entities, the establishment of this criterion lacks support from clinical or scientific data (23). Recent research findings have indicated that adopting lower standards may be more suitable, as even a 10% NED status has a notable impact on prognosis (24). As we learn more about these tumours, it increases the likelihood that formally defined thresholds will change in the future.

The naming of MiNENs has undergone many changes over the past few decades, the alterations in their terminology further emphasize the difficulties linked to MiNENs. Mixed tumours with exocrine and neuroendocrine components were first described by Cordier in 1924 (25), only a short time following the discovery of pure NE tumours in 1907 (26). Since then, a multitude of distinct designations have been employed, leading to perplexity within the medical community comprising physicians, surgeons, and pathologists. These designations include argentaffin cell adenocarcinoma, mucin-producing carcinoid, and composite carcinoid. In 2000, Capella et al. launched the initial endeavour to establish a standardized vocabulary and prognostic classification for mixed tumours occurring in the gastrointestinal tract (27). These tumours were categorized as “mixed exocrine-endocrine carcinomas” (MEECs) by the WHO Classification of Tumours of the Digestive System the same year. This classification system is used to identify tumours that have both a neuroendocrine and an exocrine component (28). The terminology encompasses the three types postulated by Lewin (19) (collision, association, and amphibious tumours); nonetheless, it is noteworthy that non-neuroendocrine carcinomas exhibiting only sporadic neuroendocrine cells were seemingly not included under this classification. The primary rationale behind this decision was based on the understanding that the existence of scattered neuroendocrine cells in adenocarcinomas or squamous cell carcinomas does not hold predictive significance. In order to underscore and strengthen this notion, a predetermined threshold of 30% was implemented for each constituent to classify the tumour as mixed. This threshold still exists, however, there is debate concerning its biological significance. The term was short-lived, in the later 2010 edition of the World Health Organization classification of tumours of the digestive system, the term “mixed adeno-neuroendocrine carcinomas (MANECs)” was officially adopted as the terminology. The selection of this phrase is likely due to the fact that mixed tumours typically comprise adenocarcinoma and NEC in the majority of instances. Although this assumption is correct, the accumulation of clinical practice has revealed that in some cases the composition of non-neuroendocrine tumours is not limited to adenocarcinomas, and that neuroendocrine tumours may also be composed of NETs, and it is clear that MANEC is applicable to the diagnosis of only a proportion of mixed tumours (29). In 2016, La Rosa et al (30) proposed the umbrella term “Mixed Neuroendocrine and Non-Neuroendocrine Neoplasms (MiNENs) “. One notable benefit of employing this terminology is its ability to encompass a wide range of entities resulting from various combinations of components. This catch-all word effectively encompasses the diverse array of entities in question. Hence, it is imperative to regard MiNENs as a conceptual classification rather than a precise diagnostic entity (31). The aforementioned terminology was first established in the fifth edition of the World Health Organization’s categorization of tumours of the digestive system in the year 2019 (16). Tumours having both NE and any NNE component can be identified using this new nomenclature; both components must account for more than 30% of the total tumour composition. Only mixed tumours originating in the digestive system were eligible for the proposal’s acceptance, nevertheless. It is important to note that “MiNENs” is a phrase used to describe a variety of mixed NE tumours; it is not a diagnosis. The term alone doesn’t provide any helpful context such as tumour composition to the oncologist, and the pathologist must ensure that these components are clearly communicated to enable the oncologist to divide up the risk and make appropriate management plans (21).

## Pathogenesis of gastric MiNENs

The mechanisms by which g-MiNENs occur are unknown. however, three primary hypotheses have been put forth: first, the neuroendocrine and non-neuroendocrine components merge after the generation of separate progenitors independently of each other, with the adenocarcinoma cells being differentiated from pluripotent stem cells, and the neuroendocrine cells originating from embryonic neuronal cells. Another theory is that these two constituents stem from shared pluripotent stem cell progenitors that experience dual phenotypic differentiation in the course of carcinogenesis. The third hypothesis posits that both components are derived from shared monoclonal cells, but the process of neuroendocrine differentiation takes place in a non-neuroendocrine phenotype as a result of the accumulation of genetic abnormalities. After the recent discovery of an overlapping mutational spectrum of the two cell varieties that comprise gastric MiNEN, this last hypothesis seems to be the most accepted today (4, 32).

Recent studies have shown that g-MiNENs are derived from a single precursor cell that is doubly differentiated after the onset of carcinogenesis (33). As demonstrated in some malignancies, poorly differentiated carcinomas may also be transformed into alternative transcriptional programmes induced by cellular plasticity, leading to neuroendocrine differentiation (34). Several studies have conducted comparisons of the mutational characteristics of NE and non-NE components, revealing a shared mutational foundation as well as distinct mutations unique to each component (9, 35).

Yeo et al, sequencing analysis of 10 g-MINENs showed that the vast majority of mutations in g-MiNENs were shared by adenocarcinoma (ADC) and NEC components, with the most common genomic variants being mutations in the TP53 gene and deletions in the ATRX gene. The NEN component in g-MiNENs carried 1.5 times more genetic variation than nNENs, most of which was copy number variation (CNV). nNENs showed copy number gain in MYC genes and copy number loss in CDKN2A and CDKN2B genes. nNENs showed exclusive CNV in RB1, RAD50, FANCD2. TERT, CCND, FGF19, FGF3, FGFR1, and RICTOR genes with exclusive CNV. Compared to NENs, nNENs had higher missense mutations in the PIK3CA and ARID1A genes. Furthermore, three unique variations in the NBN, KRAS, and CTNNB1 genes were present in nNENs (36). Ishida et al. analysed the molecular pathological features of g-MiNENs. The results showed that TP53 was the most common mutation and was more common in MiNENs than in NEC. Some patients with g-MiNENs had high levels of microsatellite instability (MSI), as well as mutations in the neuroendocrine tumour (NET)-related genes MEN1 and ATRX1. In g-MiNENs patients, mutations in TP53, APC, and ZNF521 were shared by the two histological components, whereas changes in CTNNB1, KMT2C, PTEN, and SPEN were only seen in the neuroendocrine component. To summarize, TP53 is a frequently mutated gene in gastric NECs and MiNENs, while mutations in other genes are less prevalent. This pattern of mutational spectrum in NECs and MiNENs resembles that observed in gastric adenocarcinoma (37). Additionally, chromosomal abnormalities and allelic imbalances in the NEN component increased, indicating that c-Myc and SMARCA mutations might be involved in transdifferentiation (21). This implies that MiNENs might originate as non-NE tumours and subsequently undergo transdifferentiation to develop an aggressive NEC component. Furthermore, it is possible that certain cases of g-MiNENs harbour cancer-associated disease-causing mutations that are exclusive to the NEN component, which supports the notion of a monoclonal origin and a multistep progression model for the development of g-MiNENs. In a recent molecular study of targeted DNA sequencing for gastric tumours, it was similarly found that the ADC and NEC components shared the great majority of alterations, with TP53 being the most frequently altered gene. The significant correlation between the differentially altered genes in the ADC component and the receptor tyrosine kinase signalling pathway and the significant correlation between the differentially altered genes in the NEC component and the NOTCH signalling pathway suggests that the ADC and NEC components of g-MiNENs may have originated from a common clone (35). Another finding that supports a common oncogenic pathway is the increased methylation of a portion of g-MiNENs, creating a mismatch repair-deficient phenotype. Mismatch repair errors are thought to be a potential link between MiNENs and adenocarcinoma because of the less aggressive behaviour of these tumours (35). Sun et al (38)determined and analysed data from high-resolution copy number (CN) analysis in the NEC and adenocarcinoma components of eight g-MiNENs. Both components typically showed a number of common CNVs, such as loss of FAT1 and gain of CCNE1. The possibility that morphologically diverse tumour cells have a monoclonal origin is supported by the discovery of shared CNVs in both components. CCNE1 gain and FAT1 loss may promote tumourigenesis in g-MiNENs in part by regulating cell cycle G1/S checkpoint signalling. The neuroendocrine differentiation of g-MiNENs may be aided by certain CNV and pathway abnormalities, for example, MAPK1 deletion and modified MAPK signalling pathways, that were discovered during analyses that focused on NEC components. Furthermore, our investigation revealed that compared to the adenocarcinoma component, the NEC component had higher CNV and CN loss. There appears to be high genetic heterogeneity of NEC components in g-MiNENs, as the NEC components of various cases did not cluster in hierarchical cluster analysis.

As mentioned above, based on available data from molecular and genetic investigations of gastric MiNENs, the neuroendocrine and non-neuroendocrine components appear to have a shared monoclonal origin, even though the histological origin and molecular mechanisms of g-MiNENs are still contentious. The origin of MiNENs has been connected to mutations in MiNENs-associated genes, with TP53 mutations being the most common. Furthermore, microsatellite instability and mutations in the tumour-associated genes BRAF and KRAS have been identified as probable causes of MiNENs (39, 40). The transition from non-neuroendocrine to neuroendocrine cellular phenotypes may occur more frequently than previously believed, as evidenced by the greater prevalence of chromosomal and genetic defects in neuroendocrine components compared to non-neuroendocrine components. Exclusive alterations in the neuroendocrine component carry more aberrations and allelic imbalances with more aggressive phenotypes, thus leading to the generation of a neuroendocrine component from the non-neuroendocrine component through transdifferentiation.

## Diagnosis of gastric MiNENs

**Figure 2:**
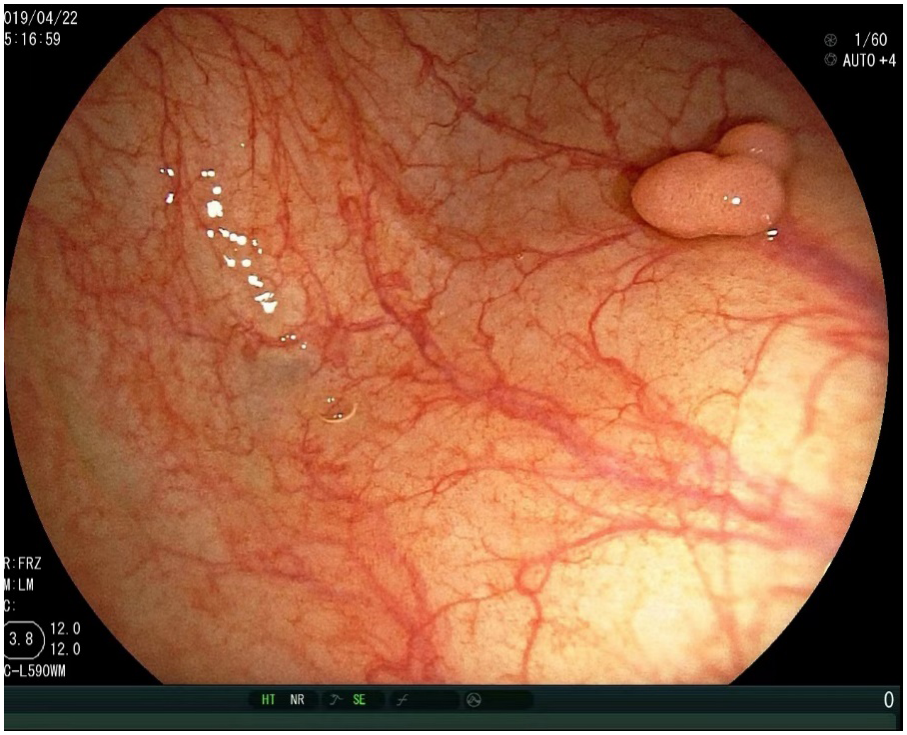
Endoscopic picture of gastric mixed neuroendocrine-non-neuroendocrine neoplasms.

Clinical manifestations and symptoms of g-MiNENs are non-specific clinical signs and symptoms similar to those of other gastric tumours, including dyspepsia, abdominal pain, nausea, vomiting and weight loss. Histologically, they consist mainly of adenocarcinoma and NEC. However, due to the heterogeneity of MiNENs, some clinical differences may exist (41). Most cases of MiNENs develop slowly and imperceptibly, and for many patients the disease is usually at an advanced stage at the time of consultation with a doctor, and most show lymph node and distant metastases (8). For individuals with G-MiNEN, upper gastrointestinal endoscopy and improved CT scanning are essential components of a comprehensive diagnostic evaluation (42). Neuroendocrine cancer pictures on CT analysis typically show isointense lesions during the pre-contrast phase, which are markedly amplified during the arterial phase following intravenous contrast administration (43, 44). With regard to endoscopy, MiNENs usually show typical manifestations: on imaging endoscopy, mucosal congestion in the area of the lesion, usually associated with central depression, is usually seen; with endoscopic magnification, central discolouration associated with visible subepithelial capillaries, forming a corkscrew pattern, is usually noted (45). Thus, MINENs usually show up on endoscopic imaging as isolated lesions, frequently in the stomach’s body or fundus, and typically measuring at least 2 centimetres in diameter (**Figure 2**) (46). Early detection and diagnosis of MiNEN are frequently crucial yet challenging. When sufficient specimens are collected following surgery, MiNENs is typically diagnosed with nonspecific symptoms of stomach cancer (at an advanced disease stage). Endoscopic observation and biopsy alone are not always sufficient to diagnose MiNENs, and extra consideration should be given to potential bias when interpreting biopsy specimens. A significant number of MiNENs cases continue to be misdiagnosed, largely as a result of inadequate histological examination parameter selection (47). Therefore, it is necessary to assess pathological specimens following the surgical excision of the complete tumour. The key to the diagnosis of MiNENs is the identification and classification of mixed tissues by histopathological analysis and immunohistochemistry (IHC) techniques on surgical specimens. According to the WHO classification, adenocarcinoma and neuroendocrine carcinoma must be present in at least 30% of each component for the pathological diagnosis of MiNENs to be made (48).

The diagnosis of MiNENs is mainly based on histopathological examination, and should be suspected when haematoxylin and eosin stains show the existence of both neuroendocrine and adenocarcinoma components (49). The next step is to confirm the diagnosis using appropriate immunohistochemical methods, detecting at least 2 of the 3 pathological neuroendocrine markers (synaptophysin, chromogranin A and CD56) (50–52). It is necessary to employ IHC techniques and the identification of morphological features for the two cellular components utilized to detect and grade neuroendocrine or non-neuroendocrine conditions. This can be challenging when both components are poorly distinguished (53). The properties of these IHC markers in gastric MINENs will be briefly described here (**Table 1**).

**Table 1:** Diagnostic markers for gastric MINENs.

G-MINENs components	Diagnostic markers
**Neuroendocrine components**	CD56, chromogranin A(CgA); synaptophysin (Syn); neuron-specificenolase (NSE); CD57; protein gene product 9.5 (PGP 9.5); insulinoma-associated protein 1 (INSM1); somatostatin receptor subtype 2A (SSTR2A); CK35BH11
**Adenocarcinoma component**	carcinoembryonic antigen (CEA); CA19-9; cytokeratins7,19; AE 1/3; CK7; CK20; caudal type homeobox 2(CDX2)

Three key neuroendocrine markers (CD56, chromogranin A, and synaptophysin) were substantially expressed in the neuroendocrine component. A variety of neuroendocrine biomarkers have been described to date, neuron-specific enolase (NSE), CD57, protein gene product 9.5 (PGP 9.5), insulinoma-associated protein 1 (INSM1), somatostatin receptor subtype 2A (SSTR2A), CK35BH11, and so on, however chromogranin A, synaptophysin, and CD56 are the most commonly used and trustworthy neuroendocrine markers (54). Adenocarcinomas express carcinoembryonic antigen (CEA), CA 19-9, cytokeratins 7, 19, AE 1/3, CK7, CK20, and caudal type homeobox 2 in the non-neuroendocrine component (4, 6).

It’s also important to keep in mind that diagnosing NEN solely through quantitative means could result in issues, especially when using IHC. Indeed, it has been reported that diagnosing MiNENs solely by quantifying IHC results can lead to terminological inconsistencies and confusion (55). Therefore, the WHO makes it very evident that these histological findings must be present in each morphological component and that IHC data alone are insufficient for the diagnosis of MiNENs (56). Another issue that should be considered is the necessity to give a diagnosis of MINENs in cases that do not receive neoadjuvant therapy. Numerous investigations have noted a potential rise in neuroendocrine cells following treatment for gastric adenocarcinoma (particularly chemotherapy) (57, 58). These tumours are not classified as MiNENs in the most recent WHO classification, and the precise mechanism underlying this behaviour is yet unclear (56).

## Staging of gastric MiNENs

Currently, there is no specific TNM staging system for gastric MiNENs, and staging criteria now depend on the assessment of size, degree of invasion, and its relationship to anatomical landmarks similar to non-NEC staging of the same site, but with differences between them that complicate assessment. For localized gastric MiNENs, endoscopic ultrasound (EUS) is recommended for staging all gastric MiNENs in addition to standard cross-sectional imaging as a complement to conventional imaging. In patients at risk for clinically significant residual or developing metastatic disease, somatostatin receptor (SSTR) imaging is necessary as a supplement to conventional imaging to confirm the presence of SSTR expression. It is recommended that the imaging of choice for SSTR-positive MiNENs is SSTR imaging plus CT and/or MRI. When clinical and laboratory findings suggest disease progression, the detection of new lesions by SSTR PET/CT associated with lesions showing stable disease on CT is considered sufficient to establish progression.

## Treatment of gastric MiNENs

Due to its low prevalence, there is still some controversy about which treatment option is the best one (38, 59). According to the WHO recommendations, MiNENs is recommended as an adenocarcinoma treatment (16). Nonetheless, according to other writers, the most aggressive histological components should determine the course of treatment (4). There are currently very few published case series on g-MiNENs, and no clinical practice guidelines have been created. Based on the Japanese categorization of gastric cancer, adjuvant chemotherapy and classical surgical resection along with lymph node dissection is the standard treatment plan for MiNENs. If there are no contraindications, aggressive surgery is the first line of treatment for g-MiNENs, and chemotherapy is the preferred course of treatment for advanced tumours that are poorly differentiated or that are growing quickly (60).

As of right now, the majority of research points to surgical excision as the main course of treatment for MiNENs in the gastrointestinal system, and surgical resection is considered to be the only curative therapy (61–63). Even in cases where patients have distant metastases, palliative treatment is still necessary. In general, surgery significantly improves survival in these patients, and patients who undergo surgical resection may have better general status and a lower burden of liver metastases. If feasible, curative surgery is essential for the treatment of MiNENs. Iwasaki, et al (11) evaluated the long-term outcome after surgery in six patients with g-MiNENs. The findings indicated that the most prevalent surgical treatment was total gastrectomy, which was followed by proximal and distal gastrectomy. 4 patients had no recurrence after resection. Following resection, the median duration of overall survival was 68.7 months. Surgical treatment is more effective in patients with MiNENs. In the context of a multimodal treatment regimen that also includes chemotherapy, aggressive resection has the potential to enhance prognosis, despite the paucity of information regarding the efficacy of surgery alone (11). Pommergaard et al. studied the efficacy of surgical treatment for g-MiNENs and found that patients treated surgically achieved a better survival benefit, with a median disease-free survival (DFS) of 12 months and a median overall survival (OS) of 32 months for patients with MINENs stage I-III. The median OS was 39 months and the median DFS was 21 months for patients with stage I–III with R0 resection. The median PFS/DFS and OS for patients with MINENs stage IV were 4 and 11 months, respectively. The median OS was 32 months and the median DFS was 6 months for patients with stage IV and R0 resection. In addition, it was shown that surgery of the primary tumour in patients with local-regional high-grade MiNENs has a better long-term outcome. Patients with primary tumours and distant metastatic disease (stage IV) may also benefit from surgical treatment (64). Laenkholm et al. found that patients with localised disease or localised disease with regional lymph node metastases had longer survival than patients with disseminated disease. Compared to patients who did not have surgery, those who had radical resection had a longer median overall survival, and the results of this study also emphasise the importance of patients undergoing surgery, and that surgery for localised disease at an early stage is more effective (7). A single-centre study that included 20 patients, 90% of whom had regional or metastatic disease. Here, R0 resection was associated with improved OS (65). This contrasts with a previous SEER database study that included 41 patients with g-MiNENs and found no prognostic impact of surgery, but did not report tumour staging data (10). Differences in outcomes may be due to the small number of patients included in the study or unequal tumour stages, and more clinical studies with large samples need to be supported in order to eliminate the possibility of surgical bias. For surgical treatment, surgical resection and lymph node dissection are the standard of care. However, the prognosis for patients undergoing these treatments remains poor, with less than 30 per cent of patients with neuroendocrine tumours being cured by surgery alone, and recurrence is relatively common (65, 66). Therefore, multimodal treatment strategies need to be developed and established to improve patient prognosis.

In many cases of MiNENs, due to their aggressive nature, MiNENs are usually diagnosed with distant metastases; therefore, in many circumstances, the combination of chemotherapy and cytotoxic medicines is a key component of the treatment plan (9). Regarding typical first-line chemotherapy for MiNENs, there is currently no agreement. Usually, systemic chemotherapy is given based on the kind of metastatic location that is there. Treatment for individuals with two components identified in the main tumour or at the metastatic location is determined by which component is the most aggressive (67). The National Comprehensive Cancer Network guidelines recommend treatments that are largely similar to protocols such as EP and IP for small cell lung cancer (68). However, CDDP+ETP is advised by the European Society for Neuroendocrine Tumours recommendations (69). According to recent research, the combination of etoposide and platinum (EP regimen) (70), or 5-fluorouracil plus irinotecan, temozolomide, or enzobiocin, is the recommended therapy for advanced MiNENs (71). However, Woo et al. (72) treated two patients with g-MiNENs with irinotecan in combination with cisplatin chemotherapy (IP regimen) and surgery, meaning that they will live for longer than three and seven years, respectively. Woo et al. (72) compared the IP with the EP regimen for the treatment of GEPNECs, the study revealed that the intervention group had a higher response rate, together with a notable duration of long-term survival spanning over three years. In the context of treating digestive neuroendocrine tumours, several studies have indicated that the occurrence of grade 4 adverse events and treatment-related fatalities was comparatively reduced in the irinotecan group as compared to the etoposide group (73–75). When evaluating progression-free survival and disease control rates, it was shown that the IP regimen exhibited a higher level of superiority compared to irinotecan monotherapy (72). This observation implies that including IP regimens into the selection of first treatment for gastric MiNENs should be given high importance. It has also been shown that cisplatin/5-FU in combination with etoposide is a common chemotherapy regimen used for the treatment of MiNENs, similar to that used for adenocarcinomas (76). The prognosis of patients with MiNENs tumours that have spread to distant places can be considerably improved via a combination of surgical removal of each metastasis and systemic treatment (77). The prolongation of progression-free survival in patients with metastatic neuroendocrine tumours can be achieved by the appropriate utilization of systemic chemotherapy in conjunction with somatostatin analogues, such as octreotide and lanreotide (8). In most cases, MiNEN expresses the somatostatin receptor on the cell surface, and octreotide and lanreotide are synthetic somatostatin analogues with longer half-lives than natural somatostatin (78). Octreotide and lanreotide were first shown to control hormone cell secretion, including inhibition of growth factors and nutrient hormones. They then also showed antiproliferative effects, such as inhibition of angiogenesis and modulation of the immune system (79). These effects are crucial for the clinical treatment of gastric MiNEN.

Given the elevated frequency of recurrence observed in these tumours, neoadjuvant chemotherapy (NAC) and adjuvant chemotherapy may provide a survival benefit for patients with g-MINENs with an acceptable level of toxicity (80). Immunotherapy by targeting immune checkpoints has been successfully used for g-MINENs (81–83). Mixed neuroendocrine and non-neuroendocrine components complicate the biological behaviour of MiNENs. Genetic analyses suggest that the two components share the same clonal origin. However, the adjuvant therapies for these two components are quite different (84). In addition, although a number of molecularly targeted drugs and immunotherapies have been initially used to treat MiNENs, evidence of efficacy is limited (1, 85, 86). Given that certain intermediate MiNENs frequently exhibit a significant presence of type 2 somatostatin receptors, it is plausible that patients with such conditions might potentially derive therapeutic benefits from the administration of long-acting somatostatin analogues and peptide-irradiated nucleotide treatment (56). Wang et al (87) evaluated the effect of NAC on these patients with g-MiNENs disease and found that patients belonging to the NAC group had superior overall survival outcomes compared to individuals who underwent surgery alone. The results of the multifactorial analysis indicated that NAC, adjuvant chemotherapy, and clinical N-staging were identified as independent variables that significantly influenced overall survival. Neoadjuvant chemotherapy may be in the process of becoming the mainstream treatment for g-MiNENs in the future.

Notwithstanding these findings, it is imperative to acknowledge the necessity for more extensive investigations in order to establish a more precise therapy approach for individuals diagnosed with MiNENs. Future research endeavours seek to elucidate the molecular susceptibility of the two constituents in instances of MiNENs in accordance with the diagnostic criteria advocated for these neoplasms. Additionally, potential efforts may be made to devise targeted therapeutic interventions against these two constituents, therefore enhancing their management and treatment. It is recommended that patients diagnosed with MiNENs undergo a comprehensive and intensive multidisciplinary oncological treatment approach. The determination of the most effective treatment strategy should be based on careful consideration of the specific components of the MiNENs.

## Prognosis of gastric MiNEN

G-MiNENs is considered to be a rare and aggressive tumour (90), and given its rarity and variability, the prognosis for MiNENs patients is still up for debate (91). Because MiNENs typically present with non-specific symptoms of gastric cancer and are typically identified at late stages of the illness, including distant metastases at the time of diagnosis, some writers have claimed that the prognosis for MiNENs is typically rather bad (89). However, some publications state that these individuals’ prognosis is comparable to that of gastric adenocarcinoma (4). In general, their prognosis is connected to aggressive behaviour, strong aggressiveness and high lymph node dissemination. In addition, the neuroendocrine component seems to be a major determinant of their clinical progression and prognosis (56, 92). Patients with MiNENs have a poor prognosis because to the tumours’ heterogeneity, fast development, high risk of lymph node metastases, and invasiveness (93). Huang et al (8) included 46 patients with GEP-MiNENs of which 35 had gastric tumours, 9 had intestinal tumours and 2 had pancreatic tumours. The median age of the patients was 66 years with a male to female ratio of 2.83. 14 patients had distant metastases, of which 13 had liver metastases. The median overall survival was 30 months. The findings indicated that many characteristics, including a Ki-67 index of ≥50%, a high proportion of neuroendocrine carcinoma, lymph node involvement, distant metastasis, and a high clinical stage, were identified as independent risk factors that significantly influenced the prognosis of individuals diagnosed with g-MiNENs.

G-MiNENs are a class of extremely dangerous tumours. The prognosis and other clinical outcomes of individuals with these tumours have not been well documented in research to far (**Table 2**). Patients with basic gastric adenocarcinoma and gastric neuroendocrine carcinoma may fare better than those with MiNENs (94, 95). However, it is unclear whether the prognosis of MiNEN of the stomach is better or worse than gastric neuroendocrine carcinoma (NEC) (96). Choi et al. (97) evaluated the clinicopathological outcomes and prognosis of patients with gastric NECs and g-MiNENs, and the study included 36 patients with gastric NECs and 85 patients with MiNENs. The findings demonstrated that whereas OS and DFS were comparable in patients with NECs and high-grade MiNENs, the DFS of patients with NECs was considerably worse than that of patients with intermediate-grade MiNENs. Cheng et al. (12), out of 56 patients with gastric NECs included, of which 14 were g-MINENs, discovered that the 5-year survival rate for non-MINENs was 57.8% and for MINENs was 50.8%. Although MINENs showed lower survival, there was no statistical difference between the two, probably due to the small sample size. Iwasaki (11) et al. included seven patients with gastric NECs and six patients with g-MiNENs in their prognosis and found that the distinction between those with MiNENs and those with NECs was not statistically significant in terms of recurrence-free period and overall survival. An analysis of the SEER database based on a large sample of 12,878 patients with NECs or MiNENs revealed no discernible variation in survival between gastrointestinal system patients with NECs and MiNENs. Patients with MiNENs who are aged 55 years or older, have been diagnosed with TNM stage III-IV, or have not undergone surgical therapy exhibit independent unfavourable prognostic markers (10). The 5-year disease-free survival (DFS) rates of 503 NECs, 401 MiNENs, and 2785 gastric adenocarcinomas were 47.5%, 51.1%, and 57.8%, respectively, according to a newly published survival comparison. In comparison to adenocarcinomas, DFS was shorter in NECs and MiNENs; however, there was no statistically significant difference between NECs and MiNENs (98).

**Table 2:** Clinical features and prognosis of gastric MiNENs.

Reference	Year	Area	n	M/F	Organ	Treatmemt	Median OS (month)
**Woo** (72)	2022	China	2	1/1	gastric	Surgery and chemotherapy	36m
**Iwasaki** (11)	2022	Japan	6	6/0	gastric	Surgery	74.7m
**Huang** (8)	2021	China	35	34/12	gastric	Surgery	30m
**Zhang** (9)	2021	China	78	–	gastric	Surgery	28.7m
**Ramos** (4)	2021	Brazil	5	4/1	gastric	Surgery and chemotherapy	37.0m
**Lou** (88)	2021	China	28	–	gastric	–	46.1m
**Chen** (55)	2019	China	10	6/4	gastric	Surgery and chemotherapy/radiotherapy/immunotherapy	18.6m
**Wu** (89)	2018	China	40	37/3	gastric	Surgery and chemotherapy	12m

Large lymph node and liver metastases are common diagnoses for G-MiNENs, and these conditions are significant risk factors for a poor prognosis (99). On the metastatic pattern, there isn’t agreement in the literature, nevertheless. The fraction of the main tumour component was used to categorize MiNENs cases in the current investigation, and each patient’s distant lesions and metastatic lymph node pathological components were evaluated. In every group, the percentage of pure NE infiltration-positive lymph nodes rose in proportion to the amount of NE component in the main tumour (100). Prior case reports have demonstrated that neuroendocrine carcinoma, as opposed to adenocarcinoma, is typically responsible for the invasion of lymph nodes and liver metastases (101). For patients with MiNENs, the fraction of components inside the original lesion offers useful information for choosing adjuvant therapy choices. In addition, the likelihood of recurrence in distant metastases is increased when there is a NE component in the original tumour (39).

Recent research has demonstrated that the more invasive neuroendocrine component has a major role in determining the prognosis of MiNENs (89). MiNENs is usually a highly aggressive tumour associated with a poor prognostic outlook. It combines high-grade neuroendocrine and non-neuroendocrine components, and clinically, there is controversy around MiNEN’s biological behaviour and prognostic variables. There are now two primary viewpoints in the field:(1) It has been suggested that the clinical course of MiNENs is determined by the volume ratio of the two components. According to Chen et al. (102), patients with MiNENs had an independent unfavourable prognostic factor in the high-volume (>50%) high-grade NE component. It was hypothesized that the primary histological component, as opposed to the histological component that makes up less than 30% of the entire tumour, influences the prognosis (103). (2) Regardless of the ratio, some studies have suggested that the more aggressive parts of the tumour should be the focus of treatment. They contend that the prognosis is impacted by a somewhat hypo-differentiated NEC component since, according to current research, the most aggressive component often determines the prognosis and malignant potential of MiNENs (104). Recent studies have shown that a higher Ki-67 index of the NEC component in gastric MiNENs is associated with a shorter OS (38), and an increased proportion of the NEC component and metastatic NEC component in lymph nodes is associated with a poor prognosis (55, 105). The proportion of each component inside the main tumour and the regional lymph node metastatic component were shown to be significantly correlated by Zhang et al. (9). A notable characteristic of the NE component was its tendency to spread, and even in cases where it made up less than 30% of the total volume of the mixed tumour, the NE component may still spread to distant metastases and affect the prognosis.

Microsatellite instability, a helpful screening sign for Lynch syndrome patients and a predictive factor for chemotherapeutic intervention, is caused by DNA mismatch repair (MMR) deficiencies (88). It is commonly recognized that a variety of genetic characteristics give important information that helps patients receive anti-tumour medication. Well-characterized oncogenes and/or their protein products, including as TP53, KRAS, BRAF, RB1, PTEN, APC, PI3KCA, and MYC, are the most frequently altered genes in MiNENs (106). Microsatellite instability (MSI) considered a putative driver of MiNENs events (39). Mutations in the mismatch repair genes, which are responsible for repairing DNA replication mistakes, are often the cause of MSI, an increase in highly repetitive DNA sequences (microsatellites) that leads to the creation of tumours (107). According to a number of studies, MSI testing can be utilized as a first screening for Lynch syndrome, a significant tumour prognostic factor, and a molecular biomarker for adjuvant chemotherapy and immunotherapy prognosis (108). In order to characterize the clinicopathological characteristics and prognosis of 44 individuals with MiNENs, Lou et al. (88) looked into their MMR status. A comprehensive immunohistochemical analysis was conducted on four mismatch repair proteins, namely MLH1, MSH2, MSH6, and PMS2. The average age at the time of diagnosis was found to be 61 years, with a majority of 75% of the patients being male. Lymph node metastases were detected in 14 individuals, accounting for 35.9% of the patient population. The predominant site of tumour localization seen in the study was the stomach, accounting for 28 individuals or 63.6% of the total cases. The analysis of clinicopathological data revealed a statistically significant correlation between mismatch repair deficit (MMR deficiency) and early TNM staging, as well as a more favourable prognosis in patients with MiNEN. Furthermore, the group with mismatch repair deficiency (dMMR) exhibited a longer overall survival (OS) compared to the group with mismatch repair proficiency (pMMR) (88).

## Conclusion

Overall, gastric MiNENs are a rare malignant tumour, accounting for less than 1% of all gastric tumours. They contain both neuroendocrine (NE) and non-neuroendocrine (NNE) components, with at least 30% of each. Furthermore, it should be noted that mixed primary tumours containing less than 30% of the original tumour volume as a neuroendocrine component are nevertheless capable of metastasizing and impacting prognosis. This observation underscores the need for additional investigation into the diagnostic criteria for MiNENs. The majority of g-MiNENs have an exocrine component of adenocarcinoma and a neuroendocrine component of NEC. Available molecular and genetic findings of g-MiNENs suggest that the neuroendocrine and non-neuroendocrine components are derived from a similar monoclonal origin. The key to the diagnosis of MiNENs is the detection of at least two of the three pathological neuroendocrine markers (synaptophysin, chromogranin A and CD56) by histopathological analyses and immunohistochemistry (IHC) techniques in surgical specimens. Radical surgery is the primary therapeutic approach after the diagnosis of g-MiNENs. In cases of poorly differentiated or fast advancing advanced tumours, chemotherapy is considered the preferred treatment modality. The prognosis of patients with g-MiNENs is influenced by several independent risk variables, including the Ki-67 index, NEC proportion, lymph node involvement, distant metastases, clinical stage, and microsatellite instability. The current literature found that g-MiNENs have a low surgical cure rate and poor prognosis, requiring multidisciplinary oncological management. There is a need to further explore the molecular basis and cytokine involvement in these tumours to identify therapeutic targets.

## Author contributions

WD: Project administration, Writing – review & editing. LL: Writing – original draft. QL: Writing – original draft. WL: Writing – original draft. ZQ: Writing – original draft. ZW: Writing – original draft. DY: Writing – review & editing.
